# Editors’ Book Table

**Published:** 1871

**Authors:** 


### Editors' Book Table.

[NOTE.—All works reviewed in the columns of the CHICAGO MEDICAL JOURNAL may be found in the extensive stock of W. B. KEEN & COOKE, whose catalogue of Medical Books will be sent to any address upon request.]

*Opium and the Opium Appetite.* By ALONZO CALKINS, M.D. Philadelphia: J. B. Lippincott & Co. 1871.

To quote the writer's own language, "is a maze as bewildering to the understanding as it is dazzling to the eye of fancy." It would be difficult to find, in medical literature at least, a more complex mass of useful and entertaining knowledge, drawn from every source, sacred and profane, medical and poetical, statistical and imaginative, from the Bible to the Arabian Nights, from the Saviour of mankind "sed longo intervallo" to the Chicago Temperance Convention. "The syllabus of topics and cases" could be equaled only by the debris of a dictionary that had passed through a threshing-machine. The work is eminently a learned one, and needs for its completion, a glossary to bring it down to the comprehension of an ordinarily educated mind.

The author has evidently mastered the whole literature of his subject, and has presented much that is valuable, while his conclusions are certainly sound and judicious, inasmuch as, deprecating all the pseudo-philanthropic expedients of the prohibitionist, he advocates such modified legislation as will protect society without infringing upon the liberty of its members, and urges the extension of the culture of the vine. While the author's compilation of facts is highly valuable, and of fancies not less entertaining, his style is deformed by verbiage and pedantry, "for he draweth out the thread of his verbosity finer than the staple of his argument."

The attic salt of classical reference imparts a delightful flavor to literature, but there may be too much salt to the porridge, and it is well to remember the rebuke to the pedantry of the Spanish monks, conveyed in the old proverb, "Sermon sin Agostino, olla sin tocino."

Into such a book it is not surprising that many errors should have crept, but some of these are inexcusable. For example, his own intelligence should have taught him, in defiance of any authority, that on the other side of "Mason and Dixon" the snuff-dippers "do" not "constitute forty" nor even four "per cent. of the population," as can be proved by authority forty times as competent as his own.

His denunciations of the deceptive description of the so-called delights of opium and hasheesh eating, as depicted in the rhapsodies of Coleridge and De Quincey, are just and true. It is not probable that they present any phenomena varying specifically from those produced by alcohol. The reviewer, although practically familiar with the first and second types of intoxication, is not with the third, and hence cannot speak positively under this head. These same illusive pictures have lured many a weak-brained enthusiast to his ruin.

It is a pity that in discussing the subject of stimulation, so-called, in its various forms, writers will not take the trouble to investigate personally the physiology of the alleged process, and no longer content themselves with assumptions upon prescript. They could thus scarcely fail to arrive at a more correct understanding of the true physiological relations of these so-called stimulants, and thereby form more reliable practical conclusions. W. H.

*Selected Obstetrical and Gynæcological Works of Sir James Y. Simpson, Bart., M.D., D. C. L., late Professor of Midwifery in the University of Edinburgh; containing the substance of his Lectures on Midwifery.* Edited by J. WATT BLACK, M.A., M.D., Physician-Accoucheur to Charing-Cross Hospital, London, etc., etc. New York: D. Appleton & Company, 549 and 551 Broadway. 1871. Pp. 852. Vol. 1.

The world-wide reputation of Professor Simpson in his chosen department must render this work an exceedingly popular one as it is certainly of marked excellence. Dr. Black, the editor, was assistant, for five years, of Prof. S., and thus acquired an intimate knowledge of his opinions, modes of practice, and writings, which enabled him to perform his part of the labor with correctness and facility.

The American publishers have produced the present volume in their usual excellent style.

*The Anatomical Remembrancer, or Complete Pocket Anatomist:* Containing a Concise Description of the Structure of the Human Body. Third Edition; with Corrections and Additions by C. E. ISAACS, M.D., Demonstrator of Anatomy in the University of New York. New York: Wm. Wood & Co., 27 Great Jones street. 1871. 12mo. Pp. 265.

The "Anatomical Remembrancer" is so well known to students that it scarcely needs to be further recommended to them. It is a pocket affair, which is characterized as well by correctness as brevity.

We chronicle its issue, however, with peculiar pleasure as showing that the great publishing house which has reproduced it, has not abandoned publication of medical books as we had surmised. Unless our memory deceives us, this is the first medical book they have published or republished the present year. It will gratify many of the profession to hear that this eminent publishing house have not as yet left the field in which they formerly achieved distinction.

*Till the Doctor Comes, and How to Help Him.* By GEORGE H. HOPE, M.D., M. R. C. S. E. Revised, with Additions, by a New York Physician. New York: G. P. Putnam & Sons, Association Building, Twenty-third street. 1871. Pp. 99.

From what attention we have been able to give this little book we are prepared to recommend it as useful for exactly what it is proposed, and physicians may safely and judiciously recommend it to their patrons.

*Practical Midwifery and Obstetrics, including Anæsthetics.* By JOHN FARMER, M.D., M.A., LL.D., etc., etc. Philadelphia: J. B. Lippincott & Co. London: J. & A. Churchill. 1871. Pp. 237.

Clear and exact, profusely and excellently illustrated, we take pleasure in commending this little book as a companion both for students and "busy practitioners."

*Hamilton on Fractures and Dislocations.* 1871. Last Edition. Our copy burned. Notice soon.

BOOKS RECEIVED.

*A Manual of Midwifery. Including the Signs and Symptoms of Pregnancy, Obstetric Operations, Diseases of the Puerperal State, etc., etc.* By ALFRED MEADOWS, M.D., Member of the Royal College of Physicians, etc., etc. First American from the Second London Edition, with numerous Illustrations. Price, $3. Philadelphia: Lindsay & Blakiston. 1871.

"Those who read the first edition of this work will bear us out in thinking that Dr. Meadows's Manual forms one of the most convenient, practical and concise books yet published on the subject. It was especially good as a student's manual, and the author has, in his second edition, sought to make it of equal value to the practitioner. The part which treats of obstetric operations has been well revised, and has received numerous additions, and the several chapters on Unnatural and Complex Labors likewise comprise much new matter. Upwards of ninety new engravings have been inserted in this edition, and, with a view to facilitate reference, the author has furnished it with a very full and complete table of contents and index. We can cordially recommend this manual as accurate and practical, and as containing in a small compass a large amount of the kind of information suitable alike to the student and practitioner.—*London Lancet*, May 6, 1871.

*Dilnberger's Handy-Book of the Treatment of Women and Children's Diseases, according to the Vienna Medical School. Part I. The Diseases of Women. Part II. The Diseases of Children.* Translated from the Second German Edition, by P. NICOL, M.D. One volume 12mo. Price, $1.75. Philadelphia: Lindsay & Blakiston. 1871.

"We noticed favorably the original of this handbook some months ago and suggested that an English translation of it, with notes showing the main points wherein the practice of our medical schools differs from that at Vienna, might be well received. Mr. Nicol has now carried out this idea, and we imagine that many practitioners will be glad to possess this little manual, which gives a large mass of practical hints respecting the treatment of diseases which probably make up the larger half of every-day practice. The translation is well and correctly performed, and the necessary explanations of references to German medicinal preparations are given with proper fullness.—*The Practitioner.*

*Acton's Functions and Disorders of the Reproductive Organs in Childhood, Youth, and Advanced Life.* By WM. ACTON, M.D. Third American from the Fifth London Edition. Octavo. Pp. 348. $3.

"We think Mr. Acton has done good service to society by grappling manfully with sexual vice, and we trust that others whose position as men of science and teachers enables them to speak with authority will assist in combating and arresting the evils which it entails. We are of the opinion that the spirit which pervades it is one that does credit equally to the head and to the heart of the author."—*The British and Foreign Medico-Chirurgical Roview.*

*Wright on Headaches.* A new Edition. Their Causes and their Cure. By HENRY G. WRIGHT, M.D., Member of the Royal College of Physicians, etc., etc. From the Fourth London Edition. Pp. 154. Price, $1.25.

*Mackenzie on Laryngeal Growths.* Growths in the Larynx, with Reports and an Analysis of 100 consecutive Cases treated by the author, and a tabular statement of every published case treated since the invention of the Laryngoscope. By MORELL MACKENZIE, M.D., author of "The Laryngoscope," "Diseases of the Throat," etc. Profusely illustrated by wood engravings and chromo-lithographs. Pp. 263. $3.

*The Physician's Prescription Book, etc., etc.* By JONATHAN PEREIRA, M.D., F.R.S. Fifteenth edition. Price, cloth, $1.25; in tucks, $1.50.

*Standard Supply Table of the Medical Department of the U. S. Army, July 1st,* 1871. From the Surgeon-General's Office.

*The Principles and Practice of Surgery.* By JOHN ASHHURST, JR., M.D., Surgeon to the Episcopal Hospital, Surgeon to the Children's Hospital, etc. Philadelphia: Henry C. Lea. 1871.

*A Treatise on Human Physiology.* Fifth Edition, revised and enlarged. By JOHN C. DALTON, M.D., Professor of Physiology and Hygiene in the College of Physicians and Surgeons, New York; Member of the New York Academy of Medicine; of the New York Pathological Society, etc., etc. Philadelphia: Henry C. Lea. 1871.

*A Practical Treatise on Bright's Disease of the Kidneys.* Second Edition. By T. GRAINGER STEWART, M.D., F. R. S. E., Fellow of the Royal College of Physicians; Physician to the Royal Infirmary; Extraordinary Member and formerly President of the Royal Medical Society of Edinburgh, etc., etc. New York: Wm. Wood & Co. 1871.

*An Introduction to Pathology and Morbid Anatomy.* By T. HENRY GREEN, M.D., Lond., Member of the Royal College of Physicians; Lecturer on Pathology and Morbid Anatomy at Charing-Cross Hospital Medical School, etc. Philadelphia: Henry C. Lea. 1871.

*Pulmonary Consumption: Its Nature, Varieties, and Treatment.* By C. J. B. WILLIAMS, M.D., F. R. S., Fellow of the Royal College of Physicians; Senior Consulting Physician to the Hospital for Consumption, Brompton; etc., and CHARLES THEODORE WILLIAMS, M.A., M.D., Oxon., Fellow of the Royal College of Physicians; Physician to the Hospital for Consumption, Brompton. Philadelphia: Henry C. Lea. 1872.

*Restorative Medicine.* An Harveian Annual Oration delivered at the Royal College of Physicians, London, on June 21, 1871. By THOMAS KING CHAMBERS, M.D., etc. With two Sequels. Philadelphia: Henry C. Lea. 1871.

*Cancer: Its Classification and Remedies.* By J. W. BRIGHT, M.D. Philadelphia: published by S. W. Butler, M.D. 1871.

*The American Practitioner:* A Monthly Journal of Medicine and Surgery. Edited by DAVID W. YANDELL, M.D., Professor of Clinical Surgery in the University of Louisville, and THEOPHILUS PARVIN, M.D., Professor of the Medical and Surgical Diseases of Women in the University of Louisville. Vols. III and IV. Louisville: John P. Morton & Co.

*Medical Thermometry, and Human Temperature.* By C. A. WUNDERLICH, Professor of Clinic at the University of Leipzig, etc., etc., and EDWARD SEGUIN, M.D. New York: William Wood & Co. 1871.

*Diseases of the Skin: the Recent Advances in their Pathology and Treatment.* Boylston Prize Essay, 1871. By B. JAY JEFFRIES, A.M., M.D. Boston: Alexander Moore. 1871.

*Modern Medical Therapeutics: A Compendium of Recent Formulæ, and Specific Therapeutical Directions.* By GEO. H. NAPHEYS, A.M., M.D., one of the editors of the "Half-Yearly Compendium of Medical Science;" of the "Physician's Annual;" late Chief of Medical Clinic of Jefferson Medical College; etc., etc. Third Edition, Revised and Improved. Philadelphia: S. W. Butler, M.D. 1871.

*First Help in Accidents and in Sickness.* Published with the recommendation of the highest medical authority. Boston: Alexander Moore. 1871.

*Treatment and Prevention of Decay of the Teeth.* A Practical and Popular Treatise. By ROBERT ARTHUR, M.D., D.D.S., author of "A Treatise on Adhesive Gold Foil;" etc., etc. Philadelphia: J. B. Lippincott & Co. 1871.

*The Young Housekeeper's Friend.* By Mrs. CORNELIUS. Revised and Enlarged. Boston: Thompson, Bigelow & Brown. 1871.

*Essentials of the Principles and Practice of Medicine.* A Handbook for Students and Practitioners. By HENRY HARTSHORNE, A.M., M.D., Professor of Hygiene in the University of Pennsylvania; Consulting Physician to the Woman's Hospital of Philadelphia; etc., etc. Third Edition, thoroughly revised. Philadelphia: Henry C. Lea. 1871.

PAMPHLETS.

*Recent Advances in Medicine, and their Influence on Therapeutics.* The Annual Address delivered before the Norfolk District Medical Society, May 10, 1871, by JOEL SEAVERNS, M.D., Boston. Published by vote of the Society.

One of the greatest necessities of modern medical education is instruction in accurate modes of thought, the development of the faculty of comparison, the art of reasoning; and for this, something more is necessary than to load the memory with specific facts from clinical statistics. Of the truth of this proposition no better evidence is needed than what would be apparent from a careful perusal of the "address" before us, which, beginning with a false antithesis, terminates with a possible inference based upon *conjectures.* The author is evidently a believer in specifics, for he thinks specifically, and seems to ignore the possibility of generalizing data.

The subject of the address, as defined by the author, is the question "whether recent advances in medical knowledge tend to strengthen a belief that by the use of remedies we may prevent, arrest or cure those functional or pathological changes in the bodily organs which constitute disease, or a belief in what is called, *par excellence*, rational medicine."

It is fair to assume the first term of the question, as the author's definition of that which is not rational medicine, although the relations of the words *functional* or *pathological* are by no means so clear to the mind of the reader as they may have been to that of the writer; and while we are perfectly willing to relinquish to him the task of defining a negation, we do not admit that rational medicine is "well enough defined" in any statement which asserts diseases to be the "passing tenants of the body," or that they occupy any such objective relations thereto.

The quotation from Professor Holmes, "that the more positive knowledge we gain, the more we incline to question all which has been received without absolute proof," would seem to involve an essential truth ineradicable by any even of the author's specifics, and we fail to see how such condition of intellectual conservatism can constitute any barrier to the progress of science.

Condemning the French school for allowing "no isolated fact, no personal experience, to contribute to the stock of knowledge, if opposed to the deductions drawn from tabulated records," the author subsequently (page 116) omits "clinical experiences," "as their results are so incapable of exact proof as to be always open to cavil."

"With this lengthy preface, then," the author "proceeds to look into the issue as between nature and art, or the expectant school and the school of treatment;" and, first, after referring cursorily to the advances in the physiology of the nervous system made by the labors of Marshall Hall, Brown-Sequard and Claude Bernard, goes on to the discussion of the "germ theory of disease," and giving Mr. Burdon Sanderson's definition of micrococci or mycrozymes, states that "microscopists, in investigating the causes of the spread of contagious and epidemic diseases, have been struck by the fact that in bodies affected by certain of these, are found various minute organisms which are *supposed* to have had a part in the production of these diseases;" and upon this *supposition*, based upon occasional observation of isolated appearances, the author proposes a reversion to a belief in specific medication.

Does the author mean to assert that these minute organisms have always and invariably been found under the circumstances referred to? If so, it would establish an *invariable* relation between the organisms and the diseases in question, or, if he could still further show that they were not only invariably found in "bodies affected by certain diseases," but were never found in bodies unaffected, he would then have determined an *essential*

relation between them, and then, and not till then, might he with logical propriety begin to test their causative relation. These conditions unfulfilled; the mere fact of the discovery of the association of microscopic organisms with contagious diseases is incidental, and from it, they may be "*supposed* to have had a part in the production of those diseases," and nothing more. Supposition is not demonstration.

Now when the further fact is asserted, of which we assume the responsibility, that microscopists, under the circumstances referred to, have not found, or have failed to find (even though looking for them, which is a strong point for the microscopist) these same organisms; or again have found organisms identical in character (Bacteria, Bacteridia, Baccilleria) in the freshly drawn urine of a patient suffering from traumatic paraplegia, in the alvine evacuations of another, the subject of diarrhea, in the exudations of aphthous stomatitis in another, in every household utensil containing water which had been allowed to stand for a few hours, in street gutters, all within a period of forty-eight hours, in localities distant from each other more than a mile, under social and sanitary conditions the most widely diverse, but always in the direction of the prevailing wind—we ask, what conclusions, bearing upon the specific relations of these organisms to epidemic disease, can be drawn? The organisms were identical; as determined by multitudinous observations, the morbid conditions, with which they were associated, different. Were they the efficient (specific) cause of the paraplegia, of the diarrhea, or of the stomatitis? Did they cause them all? or were they incidentally associated with them all, under favorable atmospheric conditions?

But granting the possibility of the production of such widely different results from one and the same specific cause, it would seem reasonable that they should be susceptible of removal by the same agent, but in the instance referred to, the diarrhea was relieved by calomel, the stomatitis by strychnia, and the paraplegia by—the sexton. We leave these two series of facts to make their own arguments.

The authority next quotes Dr. Salisbury's "belief that measles were propagated by fungi, or their spores, generated in mouldy straw;" and his subsequent "opinion that intermittent fever was the result of smilar spores which abounded in low lands,"

etc. Also, Hallier, of Vienna, who found "in the alvine discharges of a healthy child with common diarrhea, numerous moving and motionless micrococci" "which may be regarded as identical with those of cholera," as also others found in dysenteric discharges.

So, too, he finds these organisms in the stools of enteric fever, in the blood in recurrent fever, in the sputa in measles, in the pustules of cow pox and small pox, in the blood of scarlet fever, and syphilis, and in the pus from gonorrhea and soft chancre.

All of which observations prove rather too much for the germ theorist, and too little to sustain the author's views of specific medication.

Again, the experiment of Semmer, of Dorpat, quoted by Dr. Nichols, "in which true charbon was produced in a colt by the injection into its jugular veins of water containing bacterids and monococcus cells from an animal with charbon," proves "only this, and nothing more," that charbon is communicable by inoculation.

We will not attempt to follow the author farther through the fascinating field of microscopic investigation in the cultivation of the "germ theory," which he has evidently studied with care and diligence, having said enough, we think, to show its insufficiency to cover the multiple and varied conditions constituting disease, and *ex necessitate* the inefficiency of germ-destroying specifics to rectify those conditions.

In contemplating with our author the brilliant physiological discoveries of Hall, Sequard, Bernard and others, with no desire to depreciate their value, we assert that they are only factors, important factors indeed, in the problem of disease, but isolated, and valueless to the practitioner until associated with the other and varied elements which must be taken into consideration in estimating the sum total of any, even the simplest, morbid process.

Admitting that ergot of rye has the power of exciting contraction in the involuntary or unstriped muscular fibres of the uterus, etc., of what value is this knowledge, isolated from any other, for it is in this relation that the author asserts its value, for when do we ever meet a case of disease in which spasm of unstriped muscle is the only pathological factor?

Again, what practical value is it to know that "bromid of potass. is a vascular sedative, repressing local congestions of the brain and other organs," unless we know the causes (perhaps irritation of the pelvic viscera) of those congestions, and can remove them?

To some of his physiology we must demur slightly, however, *i. e.*, digitalis certainly increases cardiac action, but *does not* increase arterial tension. To stimulate the action of the heart, and of the arteries at the same time, would be to energize two antagonizing forces, which would neutralize each other, or destroy the mechanism.

Again, we cannot understand how atropia by "contracting the muscular fibres of arteries, stimulating the flow of blood in organs, can assist their functions and even produce active congestion." By contracting the calibre of arteries, the amount of blood conveyed to organs must be diminished, while the rapidity of its flow may be increased, cardiac force remaining unchanged. Moreover, practically, atropia *does not* produce active congestion, but, by contracting the calibre of the peripheral vessels, sends the blood back to the central organ, and if the heart be excited by too great repletion, there results an energetic reflex action which dilates the capiliary vessels and restores vascular equilibrium, temporarily disturbed by the atropia.

These specific effects of drugs are exceedingly valuable, as they constitute the single letters of the alphabet of the science of therapeutics, by whose combinations we hope eventually to construct a language, in which we may learn truths of a broader significance than that this or that drug "is good for" this or that symptom.

At the beginning of his address, and after having attempted to define a negation, *i. e.*, that which *is not* rational medicine, the author thinks that which *is* is "well enough defined" by the following "extract from Dr. Gibson's address on Medicine before the British Medical Association, in August, 1870, quoted by an admirer in the *Boston Medical and Surgical Journal*, of the following month."

"Diseases have," he says, "so to speak, a lifetime of their own, with its periods of growth, maturity and decline. They are the passing tenants of the body which they occupy often with great injury for a limited time. Treatment cannot change their nature, cannot expel them at once, cannot quench them, cannot materially shorten or prolong their existence." Against which definition we beg leave, in behalf of rational medicine, to protest. In order to properly define rational medicine, it is necessary first to appreciate its object and aim, which is undoubtedly identical with his own, *i. e.*, the prevention and cure of disease; and in order to define disease, it is necessary to understand its relative term, health.

Health is not an entity, but a condition of an entity, a condition characterized by perfect integrity of structure, and equilibrium of function. Any disturbance of this condition, in whatever direction, effectually destroys it, substituting therefor another condition, which is not health, it is dis-ease—ease disturbed.

Now, when we come to consider man in his threefold nature, *i. e.*, mental, moral and physical, and the complex relations which through these media he must hold with all that is outside of himself, and the still more complex correlations of that threefold nature within itself; when it is recognized that the most, apparently, trivial change in any one of these relations may be sufficient to destroy the organism; when we know that the addition of five per cent. to the amount of carbonic acid gas in the atmosphere is sufficient to produce fatal asphyxia; that a violent fit of anger may occasion apoplexy; that the over indulgence of some passion or emotion, good or bad, may result in insanity, or incurable cerebral disease; our view of the causes and phenomena of disease is extended infinitely, and we begin to appreciate the object and aim of rational medicine, and to lose confidence in our ability to unravel these complications with the assistance of drugs alone. When the overworked student comes to us, from his gloomy, ill-ventilated room, with head-ache, disordered digestion, and inability to sleep, shall we give him atropia, because it contracts the muscular fibres of arteries and diminishes the circulation in the brain, and tonics, to improve his appetite? or shall we give him these to remove the consequences of his violation of the laws of life, and at the same time give him fresh air, freedom from care and mental labor, exercise, and simple food—which course, alone, will the more rapidly restore health to that overworked brain?

To the wretched little victim of cholera infantum, breathing the foul air of a crowded tenant house, in a filthy alley, scantily fed on milk from a swill-fed cow, or a whisky-fed mother, which will soonest restore health, antiseptics, or pure milk and the air of the country or the sea-shore?

Moreover, we assert that the causes of disease are not always direct, immediate, nor simple. In considering the human organism as perverted in disease, we have not before us the simple elements of a type perverted, a special direction, and a specific agent. This were indeed a simple problem for solution, and medicine would be a purely practical art.

But when, as is really the case, the perversion which attracts our attention may be simply "the last straw which has broken the camel's back," the last one of a series of concurrent causes, accumulated during a long time preceding, perhaps a lifetime, perhaps generations, by the agency of hereditary proclivities, personal characteristics, habits and modes of life, occupations, climate and local influences, the problem becomes more complicated, more general, and defies solution by specifics.

Rational medicine proposes to consider disease and its victims in these comprehensive relations, and for its justification can point to the diminished mortality, under any and all forms of disease, to the vastly increased average longevity in communities where it has been the governing influence, and also to the very advances in special departments of knowledge, with which our learned author seems so familiar, which have been made in obedience to the comprehensive demands of rational medicine, to fill some constantly recurring want in her grand scheme of science. W. H.

*Report of Committee on Otology of Illinois State Medical Society.* By SAM'L J. JONES, M.D., Chicago,

Is confined chiefly to the progress made, more especially during the past year, in diagnosis and treatment of some of the more frequent diseases of the year. Refers to the use of the tuning-fork in determining the condition of the Eustachian Tube, and its mode of application; to chronic catarrh of the middle ear, and the mode of cleansing the middle ear by syringing the same through an incision in the meatus toward the Eustachian Tube and nares, by inclining the head. Considers also suppuration in the mastoid cells, "infantile otilis," and the relations thereto of the parasite asperquillus glaucus.

Referring to the use of electricity in the treatment of aural disease, the committee prefers (very wisely) to suspend judgment until further investigation shall have determined more distinctly the laws of its action.

We are inclined to think that the estimate of Troltsch, which appears to be approved by the committee, of the number of persons suffering from diseased hearing, as two out of every three, to be greatly exaggerated. However true it may be in Germany, it can scarcely apply to America.

The report concludes with valuable practical hints upon illumination of the ear, upon its auscultation, and upon the greater value of the information to be acquired by inspection of the living membrana tympani, than of the dead.

The report is brief, succinct and comprehensive, and constitutes a valuable addition to the very meagre literature of otology in America—qualities which might be imitated with advantage by writers of "Reports" generally. W. H.

*The Introductory Lecture to the course on Pathological Anatomy at the University of Pennsylvania.* Delivered, September 4, 1871, by JOSEPH G. RICHARDSON, M.D., Lecturer on Morbid Anatomy in the University of Pennsylvania, Microscopist to the Pennsylvania Hospital, etc. Reprinted from the *Philadelphia Medical Times.*

Comes from the pen of one of the most industrious workers, and original thinkers in the ranks of young physic—a staunch defender of the doctrine of "rational medicine," in opposition to the specificists.

The lecturer impresses in a very happy manner upon the minds of his auditors the importance of pathological knowledge to enable them to become accurate diagnosticians, and hence, successful practitioners, and then proceeds to give some original views which we will refer to later.

His definition of life is better than that of Herbert Spencer, whom he quotes, and whose circumlocutions are so vague as to lack almost entirely the characteristics of definitions. It is gratifying to perceive the lecturer's total rejection of the theory of morbid entities, in his definition of disease, as the condition of "abnormal relations," the index to which is pain.

Will not some philosophical pathologist give us a definition of Pain? It would certainly throw much light upon the whole subject of disease.

In regard to the development theory, the lecturer's position appears to be not very clearly defined. While asserting it to be utterly devoid of absolute proof, "as applied to the far distant epochs of all pre-historic times," he yet admits, that "in its limited application to minor variations, it accords remarkably with the events of our own era, daily transpiring before our eyes." Here is a palpable inconsistency. That which is true must be so absolutely, invariably and continually; if the possibility of specific evolution be admitted now, it must be admitted for all past and future time.

While the occurrence of variations in organisms under the operation of modifying external relations, is a fact demonstrated by the daily observation of all, the permanency of any of these varieties under any and all conditions is yet to be exemplified. Indeed, one of the strongest arguments against the development hypothesis is, the reversion of varieties to the original specific type, upon the withdrawal of external influences. That organisms, whether of the human, or of a more humble type, have, within the compass of our observation, reached a higher degree of perfection than they had previously manifested, is no evidence of the truth of the evolution theory, so called, but rather of the emergence of the true type into an actuality which previously existed potentially in the organism, though repressed in its manifestation by unfavorable conditions. For example, in the new-born infant, the man exists potentially, awaiting only time and favorable conditions for his evolution: there is no specific change—no development from a lower to a higher specific grade. So, also, many other organisms are found in an undeveloped condition, which, when more favorably surrounded, reach gradually the full measure of specific perfection of their own type.

In considering the law of heredity, in its relation to disease, the author perceives a constant tendency to degeneration, as a counter movement to that variation towards a higher and more perfect type, which constitutes the datum of the development theory; this tendency to degeneration he has formulated under the expression "The extinction of the unfit," and in this formula suggests the key to the various "problems of hereditary disease amongst which our life-work is cast."

From the operation of the law thus formulated, the lecturer deduces the cause of the superior health of brutes, as compared with ourselves; the "unfit" among them being rapidly extinguished, while science, as applied in medicine and collateral arts, struggles to counteract this tendency, and to perpetuate the unfit. This is undoubtedly true, and yet, at the same time, there is another end accomplished, the extinction of the unfit by the restoration of the type, which must not be lost sight of. The very existence of the data necessitating, or even justifying, the conclusion expressed in the author's formula, involves another conclusion also—that there is a limit to the possible perversions of specific type, beyond which the organism becomes unfit to represent the type, and, hence, is extinguished. This law of limitation, which is simply another mode of expressing the author's formula, "the extinction of the unfit," is that under the operation of which the type in all organized beings is preserved, and furnishes in its results one of the strongest arguments against the evolution hypothesis.

Just as varieties, when exempted from the influence of the conditions under which they originated, invariably revert to their original specific type, so morbid varieties are likewise short lived, and either revert to the normal standard, or perish.

The author's hypothesis of "*the inheritance of microscopic peculiarities* of formation," seems to be a necessary constituent of the law of heredity. As peculiarities of feature are undoubtedly transmitted to succeeding generations, so must peculiarities of cell growth and arrangement, which are only the ultimate anatomical factors of these features; but these are simply varieties originating in the influence of temporary conditions, and are extinguished so soon as the influence of these conditions is withdrawn.

We are glad to direct the attention of our readers to this little pamphlet, not only on account of the value of the specific propositions advanced therein, but as a general exponent of the doctrines of the school of "rational medicine par excellence" (to quote the sarcasm of a recent essayist). This author is not satisfied to "feel sure that" "Cod Liver Oil is good for consumption," but thinks that he will not forfeit his position as a *practical physician* if he should occasionally overlook the drug shop, and prescribe for his patient "nourishing food, pure air, carefully regulated exercise," pulmonary gymnastics, and protection from catarrhal attacks. And so think we. W. H.

*Medical Education in America: being the Annual Address read before the Massachusetts Medical Society, June* 7, 1871, by HENRY J. BIGELOW, Professor of Surgery in Harvard University. Cambridge: Welch, Bigelow, and Company, University Press. 1871.

The very high reputation achieved by the author of this Essay had induced us to look forward to its perusal with the greatest pleasure. We must confess, however, to have been somewhat disappointed at the disposition manifested in many of its expressions to alienate scientific medicine from medicine as an art, and to lose sight almost entirely of the fact that art, in whatever department of human effort, is simply and solely the superstructure upon the foundation of science. Therefore the broader and deeper the scientific foundation, the grander and more extensive the possible superstructure. Beginning by deprecating the danger of distracting the student's mind "from what is practical, useful, or even essential, by the well-meant enthusiasm of the votaries of less applicable sciences," he proceeds to inculcate as the dominant idea in his address "Utility in Medical Education." The assertion of such a proposition would seem to be superfluous, as it is hardly to be supposed that any one could pursue the study of medicine without the stimulus of such an aspiration.

The deprecation of extensive scientific culture appears to be entirely unnecessary in this essentially utilitarian age, in which short-cuts and by-ways appear to be the most popular roads to knowledge, or, rather, to its results.

Again, while it may be "fair to inveigh against quackery," it is not fair in the author to attempt to condemn as enthusiasm that which is opposed to quackery.

Should the author contemn his Parisian professor for lecturing upon the Plague because, as it could never in all probability plant its grim standard upon Boston common, a knowledge of its pathology and therapeutics would be of no utility to him? Is it not possible that among the hundreds who listened to that lecturer in Paris there might be some Oriental, whose native land was ravaged by this fell destroyer; or even some adventurous Frenchman, who purposed to utilize his scientific studies to the saving of human life in Smyrna or Aleppo, or some other plague-stricken spot of earth? Such a chance has happened to at least one American student. But our author admits that "there is a limit to this line of argument." "No student or artizan," he says, "is the worse for an outlook upon kindred arts and sciences which will help him to establish the true relations of his own, which will supply him with additional facilities and light for its pursuit, and with that training of his intellectual powers afforded by a systematic variation in their exercise."

In this admission had the author, instead of saying "no student" is the worse, said, every student is the better, he would have conceded all that the most zealous advocates of a comprehensive scientific medical education demand.

We must protest against the author's classification of the profession, into "those who are to do the daily work of medical attendance only, and those who may be expected to contribute something to the development of medical knowledge," "for each of whom a course of education is to be provided, such as will not rise above the proper requirements of the one nor fall below the just expectations of the other." Does he mean to degrade one class into mere hewers of medical wood and drawers of medical water; into mere nurses, who shall receive from the other class their contributions to medical knowledge to be utilized for the benefit of their patients? But how, we would ask, has medical knowledge been developed in the past, but by the generalization of data collected "in the daily work of medical attendance" by this very class which the author seeks to make the very Pariahs of the profession? If these then be deprived of that "training of their intellectual powers," which will make them careful observers and accurate thinkers, how are these data to be acquired, and how can those contributions to medical knowledge be made by this high caste class in the absence of material out of which they may be elaborated? He would demand from one, scientific development without data; from the other, utility in practical art without scientific principles. Further, the author holds "that, as a rule, outside of surgery and other surface work, it is the disease that turns for better or for worse, and not the physician that turns it," from which the inference is easy that he is disposed to look down from the surgical eminence which he has so justly attained, with somewhat too much of contempt upon those whose labors though perhaps less appreciable to the vulgar eye, are none the less demonstrable and positive in their results. "The balance of healthy function is disturbed; for a varying time the disturbance increases, and for another varying time it diminishes, until the balance is restored." Did the author ever see a case of ulceration and perforation of the intestine resulting in acute peritonitis, in which the patient was saved by controlling absolutely all peristaltic intestinal action until adhesion had occurred? If so, was it the "beast or the driver" that turned out? Will he assert that the duration and mortality of typhoid fever has not been materially diminished within the past twenty years by means of treatment deduced from a more accurate knowledge of its pathology?

How is every practitioner to attain that accurate and well defined knowledge of undisputed therapeutic principles and details, which the author would exact, without a thorough and comprehensive scientific medical education which he would deny him?

"He should know how to treat and of course to identify all common diseases and injuries so that health should be re-established, etc." Would it not be better, we ask, to so instruct the student in scientific principles which underlie all disease, that he should be able to identify "not by *name* perhaps," but by classification under pathological law, both "common" and uncommon "diseases"? When he has provided "fifty of these plain and competent men," he will find it difficult to provide "one who knows more."

"Whatever else it may or may not do, a medical school should aim, first, to give a plain, sound, solid education, without error if without ornament." Granted, but is this amount of acquisition possible to a student ignorant of his own mother tongue, and of the principles of general physics and chemistry? and we can point out to him many such diplomats of schools as prominent as his own.

Again, says our author, "the best practitioner is the man of soundest judgment," an aphorism we cannot gainsay; and further, with good judgment, added to industry and fair ability, you can make an excellent medical practitioner out of moderate medical acquirement, provided only it be of the right sort. What sort of a practitioner would you make with all these and the highest amount of medical acquirement possible?

"A little learning is a dangerous thing, etc." It is certainly unfair for the author to assume, as he appears to do, that judgment and scientific acquirement must necessarily be in inverse ratio to each other. Cannot a learned man have good judgment? Is it not reasonable to suppose, other things being equal, that the man of learning will have better judgment than the comparatively ignorant man? If this be not true, then why study at all? why not leave the whole field of medical practice to the "natural bone-setters," et id omne genus, who, despising book learning, rely upon judgment as their sole stock in trade?

While it is not necessary generally, in order to be a successful practitioner, to be "acquainted with every theory of fever," to "analyze" the patient "for urea," to "register him with the sphygmograph," "keep a thermometer under his armpit;" or "generate ozone in his apartment;" but we will venture to say: that the man who understands, who knows how to do all these, will in the majority of cases be the better practitioner: if not, then we should throw science where Macbeth would "throw physick"—"to the dogs."

In regard to the distinguished foreign practitioner who treated the case of diphtheria with small doses of copaiba, we cannot agree with the author that he "lacked judgment," he lacked knowledge or moral principle, or both. A little more learning would not "have displaced the landmarks of his judgment," but rather strengthened them.

Still later; says our author these remarks are not intended as a plea for mediocrity. Whatever his intention may be, they have that appearance, to the reader unacquainted with the author's professional life, which by its brilliancy certainly falsifies the tendency of this essay. And moreover they will be assumed as such a plea by half the ignoramuses in the land who affect to see better in their own professional twilight than others in the full daylight of scientific truth.

From the succeeding pages in which the writer indicates some of the subjects which should more especially occupy the student's attention, and from the accuracy and extent of the information which he demands upon those subjects, we are inclined to think that the author does not practice what he preaches. We feel sure that the following cannot illustrate either his practice or that of any honorable, conscientious physician, viz.: "The matter of prescribing in every day practice stands thus: First, does the disease, on any ground, require a prescription? Second, does the patient? If the former, let the prescription convey, with the word, the blow; but if you prescribe for the patient, and not for the disease, the prescription, then an empty word, a vox, if need be, should convey also a preterea nihil of undoubted innocence."

Again, we have never had the good fortune to see one of our accomplished author's prescriptions, but we cannot believe that "such Latin and English names as are unmistakable" are therein "promiscuously intermingled as vernacular, without regard to case as if the whole were Anglicized." And yet such are his aspirations for his students. The frequent reference which the author makes to the "limited time of the student of medicine," would permit the inference that his period of study must necessarily terminate with that of his pupilage, instead of what is most frequently true, that this marks the beginning of his intelligent study. It is very true that "In this country the question is, What is the most profitable investment of time, capital and labor?—and in answer we say that he who wishes to extract the most *money profit* out of his time, capital and labor, had better leave the study of medicine to others, and devote himself to trade or the mechanical arts, which are pecuniarily infinitely more profitable.

The author would exact from his student accurate knowledge in Anatomy, Physiology and Therapeutics, and yet revolts in disgust at the "Horrors of Vivisection," whose "recorded phenomena, stored away by the physiological inquisitor on dusty shelves, are mostly of as little present value to man, as the knowledge of a new comet, or of Tungstate of Zirconium." We confess our astronomical and chemical deficiencies to such an extent as to render us unable to appreciate the value of the latter, but we have ever been under the impression that, to Magendie and Marshall Hall, Alcock, Prochaska, Romberg, Muller, to Claude Bernard, and Brown Sequard and many others, vivisectors, we owe all that is valuable in modern physiology, or what is the same thing, in physiology—and what is there of medical science, or of medical art, that is not based upon these very physiological laws, discovered, and discovered only, by vivisection?

There are some curious inconsistencies in this pamphlet, the most striking of which is the following, which we quote as indicating ideas so totally different from the spirit of the rest of the volume, that we cannot help suspecting them to be the real ideas of the author, which he has unconsciously inadvertently allowed to escape him. "Let us have liberal education in its widest sense, the highest education possible to the whole mind, and the whole body of the largest number, everywhere—but then let us begin at the beginning, and teach the child, and not at the end;" to all of which we say most heartily, Amen.

In the portion of the address in which the machinery of education is discussed, he gives a most graphic sketch of the American Medical Association; its pretensions, its powers, and its transactions. He is evidently familiar with the subject; so familiar, that he must have been "one of them."

With regard to the relative advantages of the German or University system of education, and our own or what might be called the free college system, it would be difficult to form any comparison without taking into consideration the wide differences in the spirit and the characteristic of the people of the two nationalities. Without considering these we can only judge by the aggregate results, and so far as statistics are of any value in the account, they must be considered as indicating the practical superiority of our own over the average European practitioner. As specialists they undoubtedly excel us; as general practitioners, the balance, we believe, turns the other way.

It is gratifying, truly, to know the rapid progress which the German school has been making of late toward accuracy of thought, and experiment and vigor of deduction, and there is doubtless room for still further progress in the same direction, which we trust will be speedily realized.

And it is still more gratifying to read the author's mode of killing Huxley's protoplasm, and with it its materialistic basis.

His review of the medical department of the German University is exceedingly interesting, and will well repay for its perusal one not already familiar with its machinery. It can scarcely be disputed, however, that the boasted superiority of the German graduate over our own, is due very much more to the greater length of time devoted to study, than to any intrinsic superiority in the mode or form of teaching.

Should the present three years' term of our American Colleges be increased, as in them, to five, and a higher standard of preliminary education exacted, the result could scarcely be other than an elevation of the profession in every desirable respect, but we fail to see how that is to be attained by simply re-arranging the subjects of study, all of which are still to be comprehended within the term of three years. W. H.

*Cholera Infantum.*

One of the best, if not the very best, article upon this all important subject we have ever met, is contained in the September No. of the St. Louis Medical and Surgical Journal from the pen of our accomplished friend, Dr. William S. Edgar, of St. Louis. The article is comprehensive and at the same time concise.

Disregarding the ordinarily assumed causes of the disease, he decidedly attributes the efficient agency in the production of this malady to heat. The meteorological statistics of various European and American cities are cited comparatively with the Mortality Reports, and when collated seem to demonstrate as true, that if long continued and intense heat be not the sole, it is at least an essential factor in the concurrent causes of cholera infantum.

The author's views upon the injurious effects of a diet consisting exclusively or largely of impure milk—and under this head he includes not only that of swill-fed, but also of stall-fed cows—are most just, and deserve to be widely disseminated, and thoroughly impressed upon the minds of the profession and the public as well.

That milk may contain deleterious elements which are entirely unappreciable by any of the ordinary scientific tests, we have been long convinced; and, moreover, that nothing would contribute so largely to the reduction of infant mortality in large cities, as such municipal regulations as would positively prohibit the sale of such poisons, and secure proper food for infants.

Dr. Edgar's article includes his views of the ætiology, pathology, therapeutics and hygiene of the disease, which, as he justly remarks, destroys thousands of children annually in our large cities. It is undoubtedly more fatal in the aggregate mortality of series of years than Asiatic cholera, and yet while using every precaution to prevent the spread of the latter, we are singularly apathetic regarding the former and greater danger. W. H.

PAMPHLETS RECEIVED.

*A Review of Darwin's Theory of the Origin and Development of Man.* By JAMES B. HUNTER, M.D. Reprinted from the Journal of Psychological Medicine, July, 1871. New York: D. Appleton & Company. 1871.

*Braithwaite's Retrospect of Practical Medicine and Surgery.* Part LXIII. July. Uniform American Edition. New York: W. A. Townsend, publisher. 1871. $2.50 a year. Half-yearly parts, $1.50.

*The Half-Yearly Abstract of the Medical Sciences, etc., etc.* Vol. LIII. July, 1871. Philadelphia: Henry C. Lea. 1871. $2.50 a year. Single volume, $1.50.

*Transactions of the American Ophthalmological Society.* Eighth Annual Meeting. Newport. July, 1871.

*Transactions of the Medical Society of the State of West Virginia.* Instituted April 10th, 1867.

*The Physicians' Annual for* 1872. *A Complete Calendar for the City and Country Practitioner.* Pp. 82. Philadelphia: S. W. Butler, M.D. 1872.

*Report of Surgical Cases in the Army. Circular No.* 3. Surgeon-General's Office. Washington, Aug. 17, 1871.

*First Biennial Report of the Board of State Commissioners of Public Charities of the State of Illinois.*

*Transactions of the Second Annual Session of the Medical Society of Virginia.* 1871.

*Spectrum Analysis: Three Lectures by Professors Roscoe, Huggins, and Lockyer.* No. 7. University Series. New Haven, Conn.: Charles C. Chatfield & Co. 1872.

*Addresses at the Inauguration of Allen R. Benton, as Chancellor of the University of Nebraska.* Sept. 6th, 1871.

*The Old Franklin Almanac for* 1872.

*Transactions of the Medical Society of the State of Pennsylvania, at its Twenty-Second Annual Session.* Sixth Series. Part II.

*Remarks upon the Diagnosis of Ovarian Tumors—From Fibrocystic Tumors of the Uterus.* By CHARLES C. LEE, M.D., Surgeon to the Charity Hospital, etc.

*On Chronic Hypertrophy of the Lips.* By R. W. TAYLOR, M.D., Surgeon to the New York Dispensary Department of Venereal and Skin Diseases.

*Vick's Illustrated Catalogue and Floral Guide for* 1872. JAMES VICK, Rochester, N. Y. Profusely Illustrated. Pp. 120.

*L'Union Medicale Du Canada. Revue Medico-Chirurgicale paraissant tous le mois.* Redacteur: J. P. Rottot, M.D. Assistant Redacteurs: A. Dagenals, M.D., L. J. P. Desrosiers, M.D., Montreal. Janvier, 1872. Vol. I, No. 1. $3 par annee.

*Industrial Monthly.* A Practical Journal for Manufacturers, Mechanics, Builders, Inventors, Engineers, and Architects. With a Record of Railway Progress. Vol. I, No. 1. 1872. $1.50 a year. Single Nos. 15 cts. Issued by the Industrial Publication Company, 176 Broadway, N. Y.

*New Remedies. A Quarterly Retrospect of Therapeutics, Pharmacy, and Allied Subjects.* Edited by HORATIO C. WOOD, JR., M.D., Professor of Medical Botany, University of Pennsylvania, Physician to the Philadelphia Hospital, etc., etc. New York: Wm. Wood & Company, publishers. $2 per annum.

We have become somewhat tired of noticing new journals, which come to us long enough to get a notice and a place on our exchange list and then die out. But from the well known character of the publishers and ability of the editor, we are inclined to believe this new candidate for professional favor will meet with better fortune. The initial No. promises well.

*The Kansas Magazine.* Vol. I, No. 1. January, 1872.

This is a new secular monthly got up in about the style of the Atlantic, and well filled with original and excellent reading matter. $4 a year. Single copies, 35 cts. Address Kansas Magazine Publishing Co., Topeka, Kas.

